# Vaccine-induced NA immunity decreases viral shedding, but does not disrupt chains of airborne transmission for the 2009 pandemic H1N1 virus in ferrets

**DOI:** 10.1128/mbio.02161-24

**Published:** 2024-09-09

**Authors:** K. M. Septer, T. A. Heinly, D. G. Sim, D. R. Patel, A. E. Roder, W. Wang, M. Chung, K. E. E. Johnson, E. Ghedin, T. C. Sutton

**Affiliations:** 1 Department of Veterinary and Biomedical Sciences, Pennsylvania State University, University Park, Pennsylvania, USA; 2 Huck Institutes of Life Sciences, Pennsylvania State University, University Park, Pennsylvania, USA; 3 Emory-UGA Center of Excellence of Influenza Research and Response (CEIRR), University Park, Pennsylvania, USA; 4 Department of Biology, Pennsylvania State University, University Park, Pennsylvania, USA; 5 Systems Genomics Section, Laboratory of Parasitic Diseases, NIAID, NIH, Bethesda, Maryland, USA; Johns Hopkins University, Baltimore, Maryland, USA; St. Jude Children's Research Hospital, Memphis, Tennessee, USA

**Keywords:** influenza, influenza vaccines, airborne transmission, ferret model, neuraminidase

## Abstract

Split-virion-inactivated influenza vaccines are formulated based on viral hemagglutinin content. These vaccines also contain the viral neuraminidase (NA) protein, but NA content is not standardized and varies between manufacturers. In clinical studies and animal models, antibodies directed toward NA reduced disease severity and viral load; however, the impact of vaccine-induced NA immunity on airborne transmission of influenza A viruses is not well characterized. Therefore, we evaluated if vaccination against NA could disrupt chains of airborne transmission for the 2009 pandemic H1N1 virus in ferrets. Immunologically naïve donor ferrets were infected with the 2009 pandemic H1N1 virus and then paired in transmission cages with mock- or NA-vaccinated respiratory contacts. The mock- and NA-vaccinated animals were then monitored daily for infection, and once infected, these animals were paired with a naive secondary respiratory contact. In these studies, all mock- and NA-vaccinated animals became infected; however, NA-vaccinated animals shed significantly less virus for fewer days relative to mock-vaccinated animals. For the secondary contacts, 6/6 and 5/6 animals became infected after exposure to mock- and NA-vaccinated animals, respectively. To determine if vaccine-induced immune pressure selected for escape variants, we sequenced viruses recovered from ferrets. No mutations in NA became enriched during transmission. These findings indicate that despite reducing viral load, vaccine-induced NA immunity does not prevent infection during continuous airborne exposure and subsequent onward airborne transmission of the 2009 pandemic H1N1 virus.

**IMPORTANCE:**

In humans and animal models, immunity against neuraminidase (NA) reduces disease severity and viral replication during influenza infection. However, we have a limited understanding of the impact of NA immunity on viral transmission. Using chains of airborne transmission in ferrets as a strategy to simulate a more natural route of infection, we assessed if vaccine-induced NA immunity could disrupt transmission of the 2009 pandemic H1N1 virus. The 2009 pandemic H1N1 virus transmitted efficiently through chains of transmission in the presence of NA immunity, but NA-vaccinated animals shed significantly less virus and had accelerated viral clearance. To determine if immune pressure led to the generation of escape variants, viruses in ferret nasal wash samples were sequenced, and no mutations in NA were identified. These findings demonstrate that vaccine-induced NA immunity is not sufficient to prevent infection via airborne exposure and onward airborne transmission of the 2009 pandemic H1N1 virus.

## INTRODUCTION

Worldwide, influenza A and B viruses are estimated to infect 1 billion people annually with 3–5 million cases of severe illness and up to 500,000 deaths (1–5). Vaccination is the most effective strategy to reduce the disease burden caused by influenza infections (6), and the most widely used vaccines are split-virion inactivated vaccines (7). These vaccines consist of fragments of the viral envelope carrying the viral hemagglutinin (HA) and neuraminidase (NA) surface proteins. Human studies have shown that anti-HA antibodies, specifically a hemagglutination inhibition antibody titer of 1:40, correlate with reduced disease severity (6–9). As a result, split-virion influenza vaccines are formulated to induce immunity toward the HA and are standardized to contain 15 µg of HA for each virus strain (8, 9).

Anti-HA antibodies generated in response to vaccination or infection are strain specific. These antibodies can directly neutralize the virus, or they can exert their effect via non-neutralizing mechanisms such as antibody-dependent cellular cytotoxicity (10). Under immune pressure, the viral HA acquires point mutations at antigenic sites (i.e., antigenic drift). Due to these changes, influenza vaccines become less efficacious over time and it is necessary to regularly update seasonal influenza vaccines to match circulating strains (11, 12).

The viral NA is the second major surface glycoprotein, and it has two well-recognized functions: (i) NA cleaves sialic acids present in mucins allowing the virus to move through the mucus layer and access viral receptors on the surface of target cells, and (ii) NA cleaves sialic acids on the host cell surface during viral budding to allow progeny virions to be released (13–15). NA is a more highly conserved surface protein than HA and less prone to antigenic drift, making it a suitable vaccine target (16). While NA is present in split-virion-inactivated vaccines, the amount and quality of NA are not standardized and are highly variable between vaccine lots and manufacturers (17, 18). As a result, vaccines do not consistently induce a robust immune response to NA (19, 20). NA antibodies induced by vaccination or infection are non-neutralizing but can reduce viral replication by interfering with viral budding (16). Importantly, the presence of anti-NA antibodies due to prior infection has been shown to reduce disease severity and viral load upon re-infection in human clinical studies (21–23). In animal studies, vaccination of mice or ferrets to induce anti-NA antibodies has been shown to reduce mortality and viral load after lethal influenza virus challenge (24–26). While the role of NA immunity in reducing disease severity has been established, there has been limited research on the role of NA immunity in disrupting viral transmission. Prior studies in guinea pigs vaccinated against influenza B NA have shown that depending on the route of vaccination and viral exposure, anti-NA immunity can reduce airborne and direct contact transmission (27, 28). However, similar studies have not been performed for influenza A viruses or using the ferret model.

The ferret model of influenza infection is considered the gold standard for the evaluation of susceptibility, pathogenicity, transmission, and vaccine efficacy for influenza A viruses (29, 30). In this model, airborne transmission is typically evaluated using 1:1 (donor:recipient ratio) pairing of a virus-inoculated donor ferret [directly infected ferret (DI)] and a respiratory contact (RC). The DI animal is intranasally inoculated with 10^6^ infectious units of the virus in a 1 mL volume that introduces the virus into the nose and lungs. Twenty-hours post-inoculation, the DI and RC animals are then housed in transmission cages where the animals share the same airspace but cannot come into direct physical contact. After pairing, the ferrets are sampled every other day, usually via nasal wash or swab, to evaluate viral transmission (31, 32).

To simulate a more natural route of infection, rather than direct inoculation, we previously developed the sequential transmission model (i.e., chains of transmission) (33). In this model, DI ferrets are paired with primary respiratory contact ferrets (RC1) in transmission cages at 1 donor:1 recipient ratio (*n* = 6 pairs). Nasal wash samples are collected daily from the RC1 animals and assayed for viral RNA (vRNA) by qRT-PCR. Once an RC1 animal is confirmed to have vRNA in the nasal wash, it is moved into a new transmission cage and paired with an RC2. The RC1 and RC2 animals are then sampled every other day to evaluate onward transmission. Using this model, we previously showed the 2009 pandemic H1N1 virus transmitted efficiently from DI ferrets to five out of six RC1 animals and then onward to five out of five RC2 ferrets (33).

Therefore, to determine if NA immunity can impact transmission of influenza A viruses, we evaluated if vaccine-induced immunity against NA can disrupt sequential transmission of the 2009 pandemic H1N1 virus in ferrets (33). To achieve this goal, we first induced NA immunity via intramuscular vaccination with recombinant NA. We then performed sequential transmission studies with the 2009 pandemic H1N1 virus using mock- or NA-vaccinated animals as the RC1. The DI and RC2 animals were immunologically naïve and had no pre-existing immunity to influenza viruses. This approach was utilized to determine if vaccine-induced NA immunity alone is sufficient to (i) prevent viral infection after exposure to the 2009 pandemic H1N1 virus via respiratory contact and (ii) prevent onward airborne transmission if NA-vaccinated animals become infected.

## RESULTS

### Intramuscular vaccination induced high levels of anti-NA antibodies

To induce NA immunity, ferrets (*n* = 6) were given an intramuscular vaccine consisting of Sigma Adjuvant System adjuvant (also known as Ribi) and 50 µg of enzymatically active recombinant N1 NA. The NA content in split-virion vaccines is not well-characterized but is estimated to vary from <1.0 to 11 µg of NA per virus strain (34, 35). Given that our goal was to induce robust NA antibody titers, we used a much higher dose (i.e., 50 µg) that is similar to the amount of recombinant HA in the licensed human vaccine, FluBlok (45 µg of HA/strain) (36). This dose is also within the range used in human NA vaccine trials in which vaccination with 23.2 and 69.6 µg of NA-induced seroconversion in at least 80% of participants (37). As mock-vaccinated controls, an additional group of ferrets (*n* = 6) were given an intramuscular vaccine consisting of adjuvant mixed with phosphate-buffered saline (PBS). Animals were given three vaccinations, 28 days apart, based on prior studies (38, 39). Throughout the course of vaccination, total IgG NA binding antibody titers in serum were determined by enzyme-linked immunosorbent assay (ELISA), while NA inhibition titers were determined by enzyme-linked lectin assay (ELLA).

**Fig 1:**
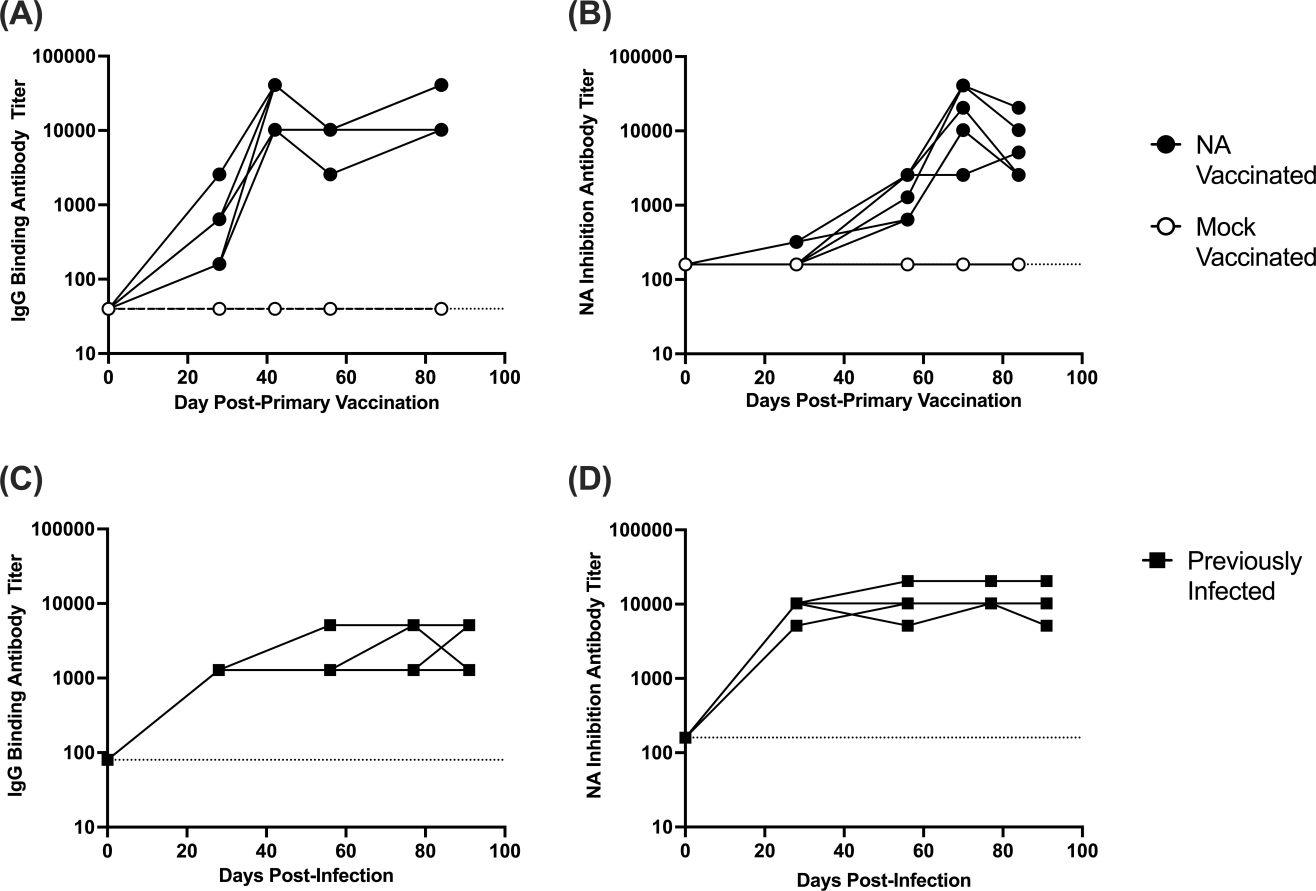
Assessment of NA binding and activity inhibiting antibodies. Ferrets (*n* = 6/group) were given a mock or recombinant NA vaccine on days 0, 28, and 56, and blood samples were collected at regular intervals to assess the antibody response to NA. Panels (**A**) and (**B**) display NA-binding IgG antibody titers determined by ELISA, and NA activity inhibiting antibody titers determined by ELLA, respectively. In a separate study (31), ferrets were infected with the 2009 pandemic H1N1 virus, and blood samples were collected for 90 days. Panels (**C**) and (**D**) show NA binding IgG antibody titers and NA activity inhibiting antibody titers in ferrets over 90 days post-infection.

In the mock-vaccinated group, none of the animals developed NA binding (Fig. 1A) or NA inhibition antibodies (Fig. 1B). In NA-vaccinated animals, an IgG-binding antibody response was detected in all animals by 28 days post-primary vaccination (Fig. 1A), while an NA inhibition response was present in one animal on day 28 and in all animals by day 56 (Fig. 1B). After secondary vaccination on day 28, the IgG binding antibody response increased to an average titer of 1:25,600 on day 42. Antibody titers then waned by two- to fourfold prior to tertiary vaccination (day 56), after which titers again increased to 1:30,720 on day 84 (Fig. 1A). NA-inhibition titers followed a similar pattern but were delayed (Fig. 1B).

As part of a separate study (31), we previously infected ferrets (*n* = 4) with 10^6^ TCID50 of A/California/07/2009 (H1N1pdm09) virus. Upon recovering from infection, these animals were maintained for 90 days, and we collected blood samples at regular intervals. When we assessed the NA-binding antibody response, NA-binding titers increased after infection and reached an average peak titer of 1:3,200 on day 77 (Fig. 1C). These titers were then maintained until the end of the study. NA inhibition titers followed a similar pattern and were similarly maintained until the end of the study (Fig. 1D). In NA-vaccinated ferrets, the levels of NA binding and NA inhibition antibody titers were comparable or higher than those in previously infected ferrets. This indicates our vaccine approach induced an antibody response comparable to that induced by viral infection.

### The 2009 pandemic H1N1 virus transmitted efficiently in chains of transmission when mock- or NA-vaccinated ferrets were the primary respiratory contact

Having confirmed our vaccination strategy induced antibodies toward NA, the vaccinated animals were then used as the RC1 in sequential transmission experiments. To initiate a chain of transmission, groups of immunologically naïve donor ferrets (DI; *n* = 6/group) were inoculated with 10^6^ TCID50 of recombinant A/California/07/2009 (H1N1pdm09) virus. Twenty-four hours post-infection, these animals were introduced into a transmission cage with mock- or NA-vaccinated animals as the RC1. The studies were set-up such that the mock- or NA-vaccinated animals were introduced into the transmission cage on day 90 post-primary vaccination. For the DI animals, nasal wash samples were collected every other day for 9 days. In the mock- and NA-vaccinated RC1 ferrets, nasal wash samples were collected daily and evaluated for the presence of viral M gene RNA by qRT-PCR. A Ct value of less than 35 was considered positive. Upon detection of infection, the RC1 animals were transferred into a new transmission cage and paired with an immunologically naïve RC2. For the second round of transmission, nasal wash samples were collected from the RC1 and RC2 animals every other day. Upon completion of the study, nasal wash samples were titrated on Madin-Darby Canine Kidney (MDCK) cells to quantify levels of replicating virus.

**Fig 2:**
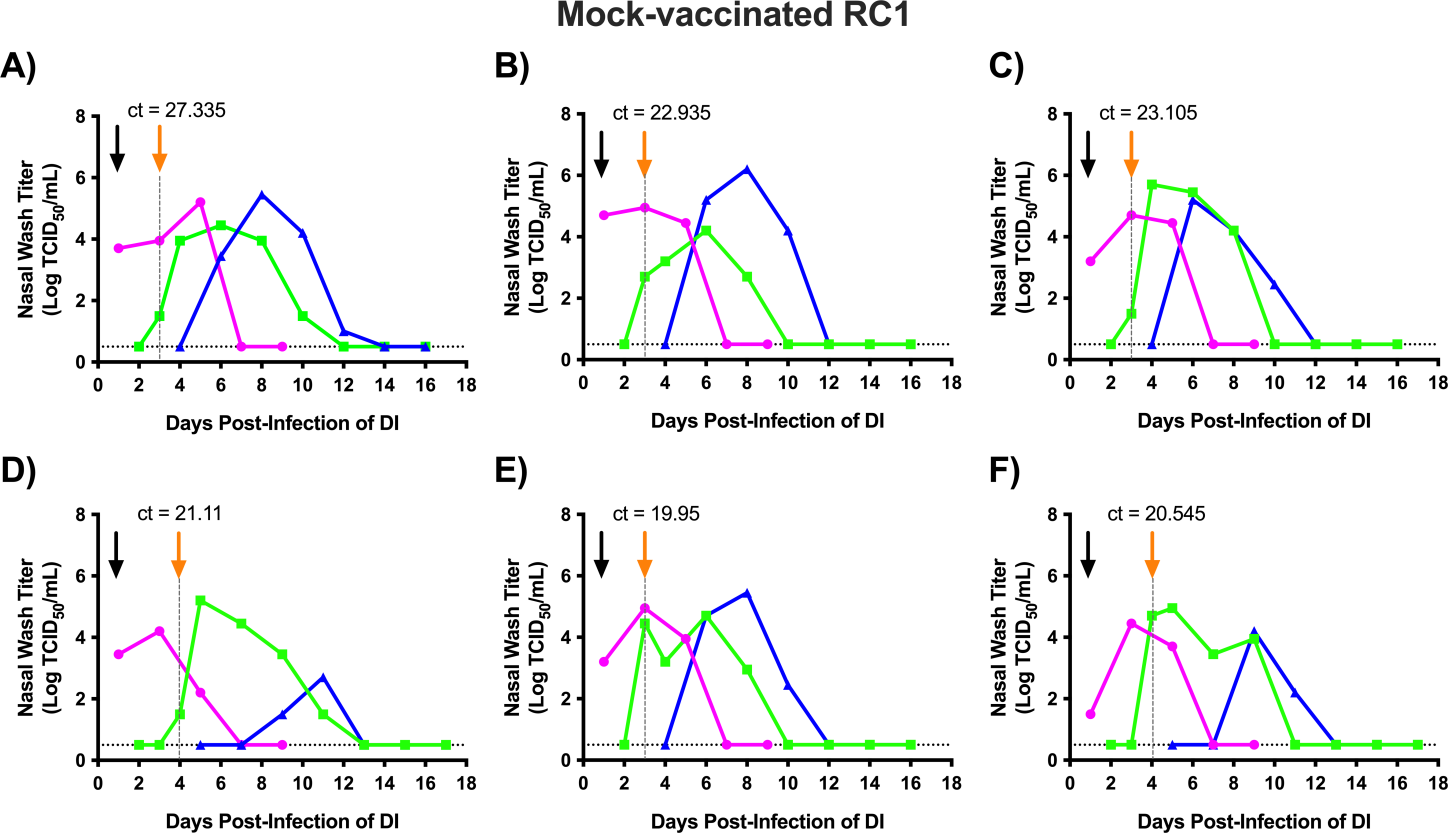
Sequential transmission of the 2009 pandemic H1N1 virus using mock-vaccinated ferrets as the RC1. Six ferrets designated as directly inoculated (DI) were intranasally infected with 1 × 10^6^ TCID_50_ of recombinant A/California/07/2009 (H1N1pdm09) virus. Twenty-four hours later, each DI animal was introduced into a transmission cage with a mock-vaccinated respiratory contact (Mock RC1) ferret. Nasal wash samples were collected daily from the Mock RC1 and assayed for evidence of viral infection by qRT-PCR. When the RC1 ferret became infected, it was housed with a new respiratory contact (RC2). Each panel (**A and F**) represents an independent replicate of DI, Mock RC1, and RC2 (i.e., ferret trio). Magenta, green, and blue lines represent the nasal wash titers for the DI, Mock RC1, and RC2 ferrets, respectively. The black arrow denotes the day the DI and Mock RC1 were paired, and the orange arrow and dashed line indicate when the Mock RC1 and RC2 ferrets were paired. Ct values for RC1 animals on the day that RC1 and RC2 animals were paired are provided above the orange arrows. Nasal wash samples were titrated on MDCK cells, and the results are expressed as log_10_ TCID_50_/mL of nasal wash. The dashed horizontal line denotes the limit of detection (0.5 Log TCID50/mL).

We initially performed transmission chain experiments using mock-vaccinated ferrets as the RC1 animals (Mock RC1; Fig. 2). After virus inoculation, all DI animals became infected and were shedding virus on day 1 post-infection. On day 3 post-infection of DI ferrets (*p.i.d*; day 2 post-contact), four of six mock-vaccinated RC1 animals (Mock RC1) were PCR positive for influenza infection (Fig. 2A through C and E, orange arrow), and vRNA was detected in the remaining two Mock RC1 ferrets on day 4 *p*.*i.d* (Fig. 2D and F). For the Mock RC1 animals, when vRNA was first detected in the nasal wash, replicating virus was also recovered (Fig. 2). After becoming infected, all the Mock RC1 ferrets showed evidence of productive viral infection with infectious titers peaking above 10^4^ TCID50/mL of nasal wash and then declining to undetectable levels by day 13 *p*.*i.d*.

Following the introduction of infected Mock RC1 animals into transmission cages with naïve RC2 animals, six of six RC2 ferrets (100%) became infected within 5 days of exposure and shed infectious virus. In the RC2 ferrets, four of six animals shed at least 10^5^ TCID50/mL of virus in the nasal wash, while the remaining two animals shed lower titers that peaked between 10^2^ and 10^4^ TCID50/mL (Fig. 2D and F). Weight loss and temperature changes varied among animals; however, no significant differences in weight loss or temperatures were detected between DI, Mock RC1, and RC2 groups (Fig. S1; Table S1). Upon completion of the study, all DI, Mock RC1, and RC2 animals developed HI titers against the 2009 pandemic H1N1 virus (Table 1), confirming all the animals (100%) became infected. Combined, the viral titer and seroconversion findings show the 2009 pandemic H1N1 virus transmitted efficiently in chains of transmission in ferrets without pre-existing immunity.

**TABLE 1:** Antibody titers in ferrets upon completion of transmission chain experiments

Transmission chain	Trio	Hemagglutination inhibition titer (reciprocal titer)		
		Donor	RC1	RC2
Mock-vaccinated RC1	1	1,280	1,280	640
	2	1,280	1,280	1,280
	3	2,560	640	160
	4	2,560	320	160
	5	640	160	320
	6	1,280	2,560	2,560
NA-vaccinated RC1	1	2,560	1,280	1,280
	2	2,560	2,560	1,280
	3	2,560	640	<1:20^*a*^
	4	1,280	1,280	1,280
	5	2,560	2,560	5,120
	6	640	5,120	10,240

^*a*^ Limit of detection is 20.

**Fig 3:**
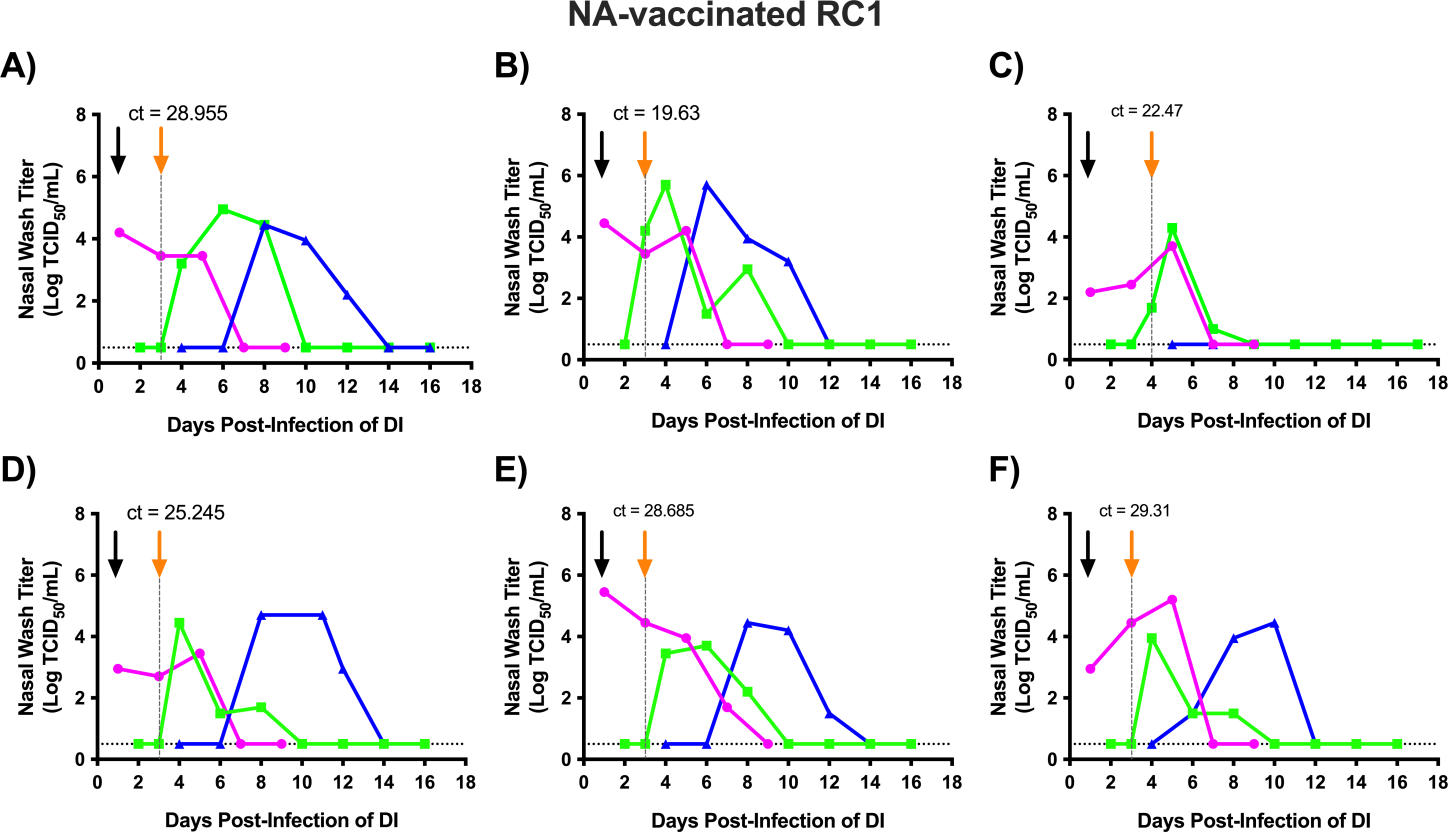
Sequential transmission of the 2009 pandemic H1N1 virus using NA-vaccinated ferrets as the RC1. Six ferrets designated as directly inoculated (DI) were intranasally infected with 1 × 10^6^ TCID_50_ of recombinant A/California/07/2009 (H1N1pdm09) virus. Twenty-four hours later, each DI animal was introduced into a transmission cage with an NA-vaccinated respiratory contact (NA RC1) ferret. Nasal wash samples were collected daily from the NA RC1 and assayed for evidence of viral infection by qRT-PCR. When the NA RC1 ferret became infected, it was housed with a new respiratory contact (RC2). Each panel (**A–F**) represents an independent replicate of DI, NA RC1, and RC2 (i.e., ferret trio). Magenta, green, and blue lines represent the nasal wash titers for the DI, NA RC1, and RC2 ferrets, respectively. The black arrow denotes the day the DI and NA RC1 were paired, and the orange arrow and dashed line indicate when the NA RC1 and RC2 ferrets were paired. Ct values for RC1 animals on the day that RC1 and RC2 animals were paired are provided above the orange arrows. Nasal wash samples were titrated on MDCK cells, and the results are expressed as log_10_ TCID_50_/mL of nasal wash. The dashed horizontal line denotes the limit of detection (0.5 Log TCID50/mL).

Next, we proceeded to evaluate transmission in the presence of NA immunity. The experimental set-up was the same as for the Mock RC1 animals, except we used NA-vaccinated RC1 (NA RC1) animals. After virus inoculation, all (100%) DI animals became productively infected and shed virus by day 1 post-infection (Fig. 3). For the NA RC1 ferrets, vRNA was detected in the nasal wash of five animals on day 3 *p*.*i.d* (day 2 post-contact), and vRNA was detected in the remaining ferret the following day (Fig. 3C). In contrast to the findings for Mock RC1 ferrets in which vRNA and infectious virus were detected at the same time, in four out of six NA RC1 animals vRNA was detected on day 3 *p*.*i.d*, but infectious virus was not recovered until day 4 *p*.*i.d* (Fig. 3A, D, E and F). For the remaining two NA RC1 ferrets, vRNA was detected on the same day when infectious virus was recovered (Fig. 3B and C). Average Ct values for Mock RC1 and NA RC1 were 22.5 and 27.72 (95% CI −1.166 ± 7.605), respectively (Fig. 2 and 3). Ct values on the day RC1 animals were transferred and paired with RC2 animals were not significantly different between Mock RC1 and NA RC1 animals (*P* = 0.24).

All NA RC1 animals [six out of six (100%)] became productively infected, and four animals had viral titers that reached 10^4^ TCID50/mL (Fig. 3A through D). The other two NA RC1 animals shed virus to peak titers of 10^3.7^ and 10^3.95^ TCID50/mL (Fig. 3E and F, respectively). After being paired with an RC2 ferret, NA RC1 animals transmitted the virus to five out of six (83%) contacts. The infected RC2 animals had viral titers that peaked near or above 10^4^ TCID_50_/mL. All animals that shed infectious virus seroconverted by HI assay (Table 1). The one RC2 animal that did not shed infectious virus also did not seroconvert. Therefore, 100% of NA RC1 animals and 83% of RC2 animals became infected via the airborne route. Similar to chains of transmission with Mock RC1 ferrets, weight loss and changes in temperature varied; however, no significant differences in weight loss or temperature change were present between the DI, NA RC1, and RC2 groups (Fig. S1B). Moreover, there were no significant differences in peak weight loss when the DI, RC1, and RC2 groups were compared between the mock- and NA-vaccinated transmission chain experiments (Fig. S1).

### Vaccine-induced NA immunity did not reduce peak titers but reduced viral shedding and accelerated viral clearance

**Fig 4:**
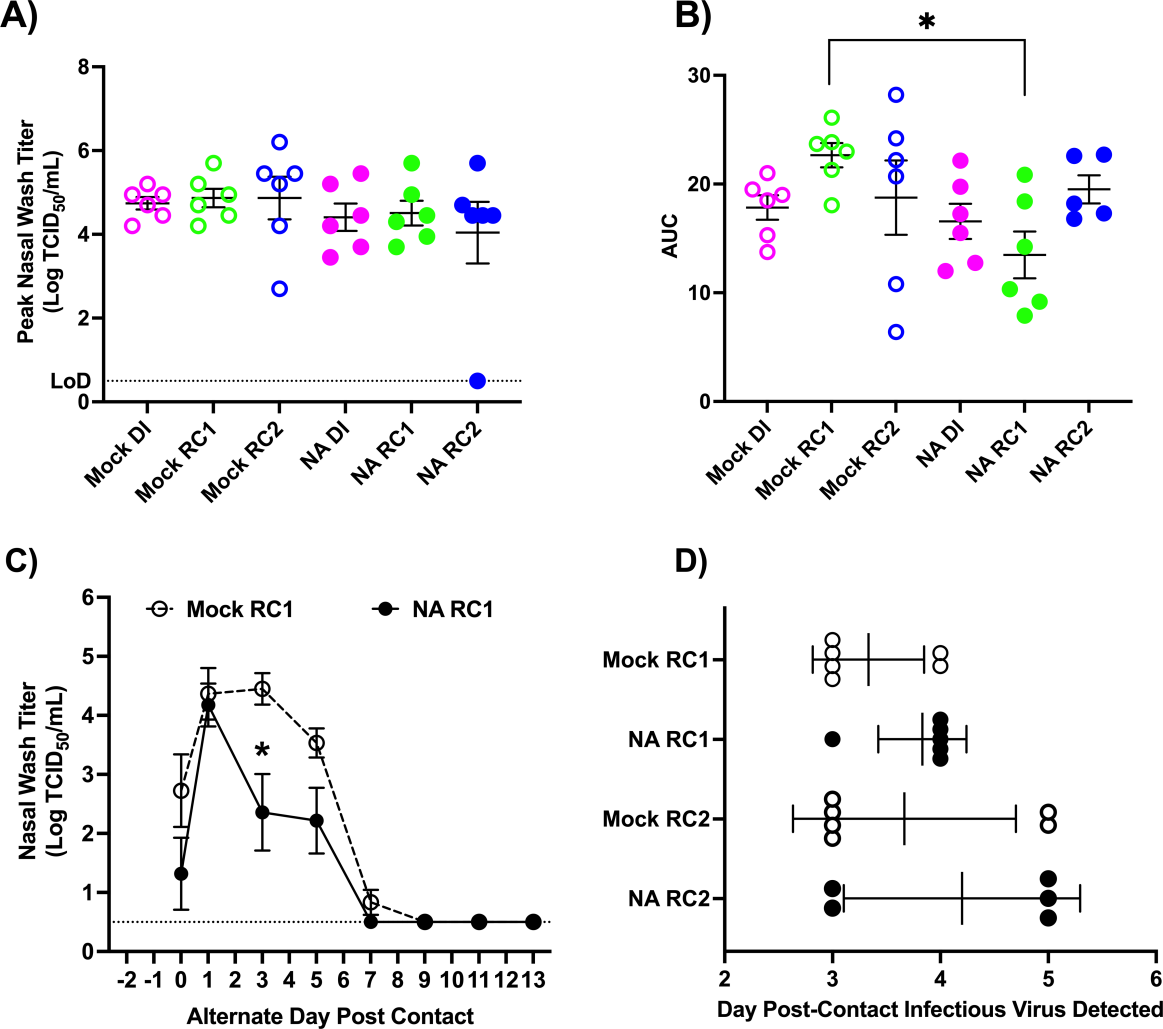
Analyses of viral shedding kinetics for transmission chain experiments using mock- and NA-vaccinated RC1 ferrets. To determine if vaccine-induced NA immunity altered viral shedding kinetics several analyses were performed. Shown in (**A**) and (**B**) are peak nasal wash titers and total viral shedding assessed by area under the curve analysis for ferrets in each experimental group, respectively. To assess if NA immunity altered the progression of viral replication, in (**C**), viral shedding curves for Mock RC1 and NA RC1 ferrets were overlaid with day 0 being the first-day vRNA was detected in the nasal wash. Shown are average titers at each timepoint in each group. Panel (**D**) displays days post-contact that ferrets in Mock RC1, NA RC1, Mock RC2, and NA RC2 groups began shedding virus after respiratory contact exposure to an infected animal. *significantly different *P* < 0.05.

While NA immunity did not disrupt chains of transmission, we further analyzed the kinetics of viral shedding to determine if NA immunity modulated viral replication. We first evaluated peak nasal wash titers for different groups of animals. Animals were grouped as follows: DI for mock-vaccinated RC1 (Mock DI), mock-vaccinated RC1 (Mock RC1), RC2 exposed to mock-vaccinated RC1 (Mock RC2), DI for NA-vaccinated RC1 (NA DI), NA-vaccinated RC1 (NA RC1), and RC2 exposed to NA-vaccinated RC1 (NA RC2). As shown in Fig. 4A, there were no significant differences in peak nasal wash titers across different groups of animals.

We then compared the total amount of virus shed from each animal post-infection using area under the curve (AUC) analyses. For the Mock DI and NA DI, and the Mock RC2 and NA RC2, there were no significant differences in the amount of virus shed; however, the NA RC1 animals shed significantly less virus overall than Mock RC1 animals (Fig. 4B). We next sought to determine if NA immunity enhanced viral clearance. To perform this comparison, we overlaid shedding curves for Mock RC1 and NA RC1 animals by setting the first-day vRNA was detected in the nose as day 0 (Fig. 4C). Consistent with our earlier analysis, both Mock RC1 and NA RC1 animals had similar peak titers of infectious virus; however, the NA RC1 animals had significantly lower titers in their nose on day 3 compared to the Mock RC1 ferrets (Fig. 4C). Viral titers were also reduced on day 5 in the NA RC1 group compared to the Mock RC1 group; however, the difference was not significant (*P* < 0.06). In addition, all the NA RC1 ferrets cleared the virus by day 7, while two out of six Mock RC1 animals were still shedding virus at this time point. This indicates NA RC1 animals cleared virus from the nose more quickly than Mock RC1 animals and more effectively controlled viral replication.

We next evaluated if there were differences in the onset of viral shedding for the Mock RC1 and NA RC1 animals and their respective RC2 ferrets. The results are shown in Fig. 4D. Infectious virus was recovered from 4/6 Mock RC1 animals 3 days after exposure to donors, and 2/6 Mock RC1 animals 4 days after exposure. For the NA RC1 ferrets, 1/6 animals shed infectious virus 3 days after exposure to an infected donor, and the remaining 5/6 animals started shedding virus 4 days after exposure. While this indicates a trend toward delayed onset of infection, this difference was not significant most likely due to a limited sample size. When we looked at the onset of shedding for the RC2 animals, similar proportions of Mock RC2 and NA RC2 animals started shedding virus 3 and 5 days after exposure to infected donors (Fig. 4D). This indicates that while NA RC1 animals shed less virus than mock-vaccinated RC1 animals, there were no significant delays in onward transmission.

Collectively, these analyses show NA immunity reduced the total amount of virus shed and induced more rapid clearance of the virus from the nose of infected animals. NA immunity may also have delayed the onset of viral shedding after exposure to an infected donor. However, NA immunity did not reduce peak viral titers or disrupt chains of airborne transmission.

### Numerous viral variants were identified in chains of transmission, but no major variants in NA were found in the presence of NA immunity

**Fig 5:**
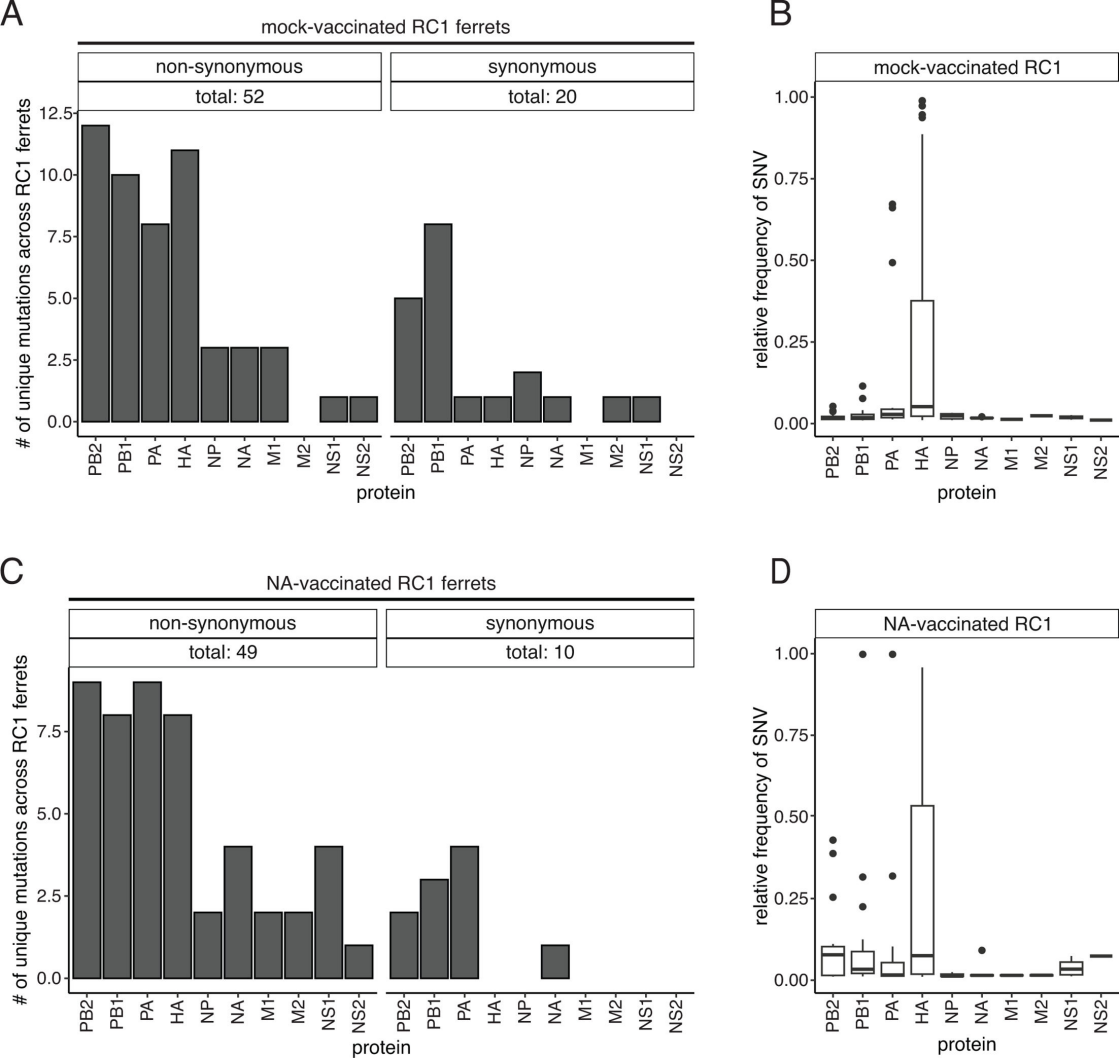
Influenza A single nucleotide variants (SNVs) in mock- and NA-vaccinated RC1 ferret nasal wash samples. (**A**) The number of unique non-synonymous (left) and synonymous (right) SNVs found in each protein (x-axis) across all mock-vaccinated RC1 ferret nasal wash samples. (**B**) Boxplots representing the median and interquartile range of the relative frequency of SNVs (y-axis) identified in the mock-vaccinated RC1 nasal wash samples. Outliers are depicted as individual points. (**C**) The number of unique non-synonymous (left) and synonymous (right) SNVs found in each protein (x-axis) across all NA-vaccinated RC1 ferret samples. (**D**) Boxplots representing the median and interquartile range of the relative frequency of SNVs (y-axis) identified in the NA-vaccinated RC1 nasal wash samples. Outliers are depicted as individual points.

**Fig 6:**
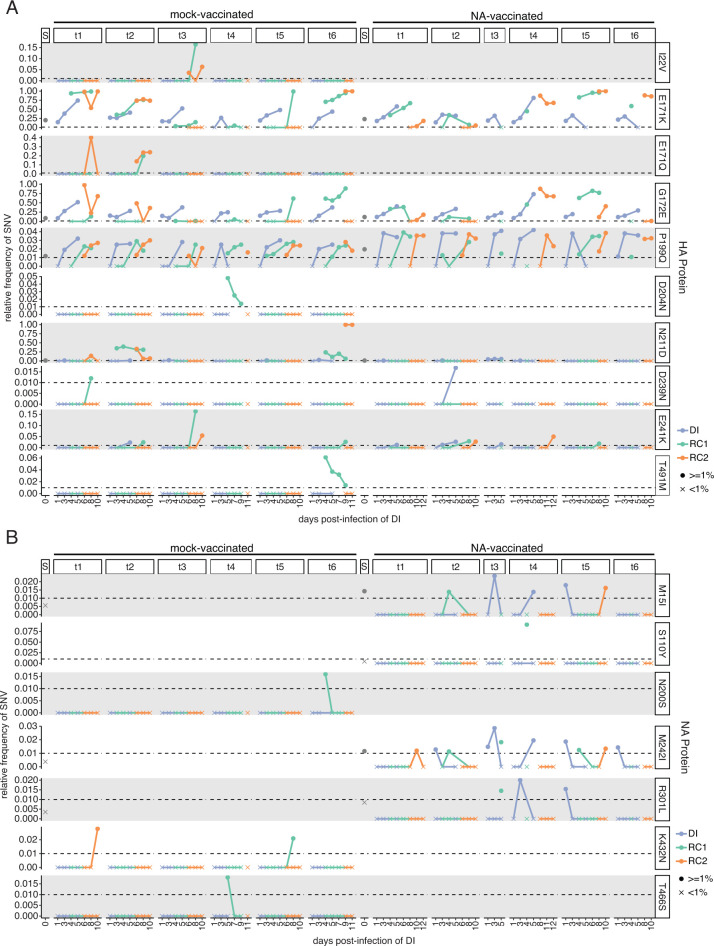
Non-synonymous mutations in the HA and NA proteins identified in two or more ferrets during sequential transmission. The displayed mutations were selected based on the criteria of being non-synonymous and occurring at frequencies of 1% (0.01) or higher in at least two samples, one of which had to be an RC1 sample collection. The relative frequency (y-axis) of (**A**) HA SNVs and (**B**) NA SNVs across the days of the experiment (x-axis). For both plots, data are grouped by the six mock-vaccinated and six NA-vaccinated transmission chains (t1–t6, across) and variants (down). The S column heading denotes the frequency of a variant in the viral stock used for infection. The point shape depicts if the variant is ≥1% (0.01, circle) or <1% (0.01, “X”) in the sample. The color of each point and line indicates the ferret in each transmission pair. DI = directly infected, RC1 = respiratory contact 1, and RC2 = respiratory contact 2. If the mutation is not observed in any of the six transmission chains for a given vaccination group, the plots will be empty. HA mutations are based on numbering from the H1 start codon.

To determine if vaccine-induced NA immunity led to the emergence of escape mutants, we sequenced viruses in all nasal wash samples with a titer greater than 10^2^ TCID50/mL. Using whole-genome sequencing, all major (≥50%) and minor variants (1.0%–49.99%) were identified. A total of 52 non-synonymous mutations and 20 synonymous mutations were identified across the six Mock RC1 ferrets (Fig. 5A). Non-synonymous mutations (≥1%) were present in all eight segments. Approximately 51% of the mutations found in the Mock RC1 ferrets were found exclusively in a single infected ferret, with only seven [HA: E171K, G172E, P199Q, N211D, E241K, PB1: V609I, and PA: G627R (Fig. 6A, left side, H1 numbering from the start codon, and Fig. S3 and S4)] and nine (HA: I22V, E171K, E171Q, G172E, P199Q, N211D, E241K, PB2: D253N, and PA: S250A) mutations shared across the DI-RC1 and RC1-RC2 pairs, respectively (Fig. 6A; Fig. S2 to S5). The most notable of which was the E171K mutation in the HA, which was present in all donor ferrets, and in these animals, the mutation increased in frequency in each successive nasal wash sample (Fig. 6A). The HA E171K mutation was then identified in five out of six Mock RC1 ferrets, and in three of these animals the frequency increased with each successive nasal wash to >75% (Fig. 6A). In each trio of ferrets in which the mutation became enriched, the mutation was subsequently identified in viruses recovered from the RC2 ferret. It is important to note that in Trio 5, the mutation was identified in one out of three nasal wash samples for the Mock RC1 ferret, and it was present at a frequency of 99%; however, this mutation was not detected in the RC2 ferret. The enrichment of HA E171K in three out of six transmission chains suggests the mutation may confer some selective advantage in ferrets.

Although not consistently enriched throughout the six Mock RC1 transmission chains (ferret trios), the mutation HA G172E was present in 11 of 18 animals. Both the HA E171K and G172E mutations are located in the Sa antigenic site of the HA, and the G172E mutation is linked to immune escape (40–42). These mutations were both present in the inoculum with frequencies of 19.2% and 8.3%, respectively. Upon completing our sequence analysis, we reviewed the sequence of the HA reverse genetic plasmid used to generate the recombinant A/California/07/2009 (H1N1pdm09) virus. This plasmid encodes HA 171K and 172G (154K and 155G in H3 numbering, respectively) (43). These findings indicate that during virus rescue and generation of a virus stock, the HA 171E mutation emerged and became the major variant; however, upon introduction of the virus into ferrets, the wild-type HA 171K mutation was selected. The HA 172E mutation also arose during viral rescue and generation of a virus stock; however, this mutation was not consistently selected in ferrets. For the other non-synonymous mutations that were identified, none of the mutations consistently increased in frequency in the transmission chains. Across the Mock RC1 ferrets, the PB2, HA, and PB1 genes had the highest numbers of non-synonymous mutations with 12, 10, and 11 each, respectively (Fig. 5A). Mutations found in the HA were also present at higher frequencies than non-synonymous mutations found in the other genes (Fig. 5B). In the NA gene, two non-synonymous mutations (NA: S89F and K432N) were identified as minor variants. NA S89F was present in one DI (not shown), and NA K432N was present in two animals, a Mock RC1 and an RC2 animal in separate trios (Fig. 6B). As neither mutation reached frequencies greater than 3% in these animals and was not transmitted onward, it is unlikely these mutations confer a fitness advantage.

Using the same approach, we sequenced ferret nasal washes from chains of transmission using NA RC1 ferrets. Forty-nine non-synonymous and 10 synonymous mutations were identified across the NA RC1 ferrets (Fig. 5C). Non-synonymous mutations were present in all eight gene segments, while synonymous mutations were only found in the PB2, PB1, PA, and NA (Fig. 5C). Sixteen unique non-synonymous mutations were shared across the DI-RC1 transmission pairs, while 12 were found to be shared between the RC1 and RC2 pairs (Fig. 6A and B; Figs S2 through S5). One non-synonymous mutation in the NA (NA S110Y) was present at a frequency of 9.1% in a single NA RC1 on one day (pair 4 and day 4; Fig. 6B), and four mutations were present in more than one sample in the NA but remained at frequencies <2% [non-synonymous: NA: M15I, M242I, and R301L (Fig. 6B) synonymous: NA C1332T (not shown)], indicating NA immunity did not lead to the generation of escape mutants. Again, the HA E171K mutation was present in all trios and became enriched during transmission in three of six trios (Fig. 6A). HA G172E was present in viruses recovered from 11 of 18 animals, but this mutation did not increase in frequency in any ferret trio. The PB2 and PA had the most non-synonymous mutations, with nine each, followed by the PB1 and HA genes, with eight (Fig. 5C); however, mutations in HA had the highest frequencies (Fig. 5D). Synonymous changes were most common in the PA gene, in which we identified four different mutations (Fig. 5C). Other than the HA E171K mutation, no other mutations increased in frequency through chains of transmission.

## DISCUSSION

In this study, we evaluated if NA antibodies induced by vaccination could impede airborne transmission of the 2009 pandemic H1N1 influenza A virus. To induce antibodies toward the NA, ferrets were vaccinated with recombinant NA, and we found that after three vaccinations, high titers of anti-NA IgG binding antibodies with high inhibition activity were present in the serum (Fig. 1A and B). By comparing the antibody response induced by vaccination to that induced by infection, we show our vaccine approach induced comparable levels of serum IgG antibodies with similar NA inhibition activity to those induced by infection (Fig. 1C and D).

To evaluate airborne transmission in ferrets, the standard approach is to infect a donor ferret with a high virus dose (≥10^6^ infectious units) in an inoculation volume that delivers virus to both the nose and lungs (44). This donor ferret is then paired with a respiratory contact animal for 10–14 days. During this time, nasal wash samples are collected and assayed for infectious virus to detect transmission to the recipient. To evaluate if vaccination can prevent transmission, vaccinated animals are often used as the donor ferrets (45–47); however, this route and dose of virus challenge is not representative of a naturally acquired infection. To address this pitfall, we previously developed the sequential model of airborne transmission which models chains of airborne transmission (33). In this model, an infected donor ferret (DI) is used to infect a primary respiratory contact (RC1), and then the primary contact is used as the donor to a second respiratory contact (RC2). Therefore, to test if vaccine-induced immunity toward NA can impede airborne transmission, we used mock- or NA-vaccinated animals as the RC1 in transmission chain studies with the 2009 pandemic H1N1 virus. We used immunologically naïve DI and RC2 ferrets, as this permitted direct assessment of whether NA immunity could disrupt a chain of transmission.

Using this approach, we found that the 2009 pandemic H1N1 virus transmitted efficiently in chains of transmission using Mock RC1 animals. Infected DI ferrets transmitted the virus to 100% of Mock RC1 animals, and these animals transmitted the virus to 100% of the RC2 ferrets (Fig. 2). This is consistent with our previous findings that evaluated sequential transmission of the 2009 pandemic H1N1 virus in immunologically naïve ferrets (33). When we performed these studies with NA RC1 animals, 100% of the NA RC1 animals became infected, and from these animals, five out of six or 83% of the RC2 ferrets became infected (Fig. 3). While this is a decrease in transmission to the RC2 ferrets relative to the Mock RC1 studies, the 2009 pandemic H1N1 virus still transmitted with high efficiency to the RC2 animals, and this variation in transmission is within normal variation observed in the sequential transmission model (33). Therefore, vaccine-induced immunity toward NA did not disrupt chains of airborne transmission. However, NA RC1 ferrets shed significantly less virus overall and controlled virus replication in the nose more effectively than Mock RC1 animals (Fig. 4). These findings indicate that the NA antibodies produced in response to vaccination were capable of limiting virus replication in the upper respiratory tract, but this reduction was not sufficient to limit airborne transmission.

To evaluate if vaccination against NA was driving viral evolution, we performed whole-genome sequencing analyses. No mutations in NA became enriched in chains of transmission with Mock or NA RC1 animals. This indicates that NA immunity did not result in the emergence of vaccine escape mutants. One mutation, HA E171K, became enriched throughout some trios (6/12 trios) in chains of transmission with Mock and NA RC1 animals. A second mutation, HA G172E, was also commonly found in chains of transmission but did not become enriched during transmission between ferrets. These mutations were both present in the inoculum (19.2% and 8.3%, respectively). Upon reviewing the HA reverse genetic plasmid sequence that was used to generate the recombinant A/California/07/2009 (H1N1pdm09) virus, the plasmid encodes HA 171K and 172G (154K and 155G in H3 numbering, respectively) (43). Therefore, it appears that during virus rescue and subsequent culture in MDCK cells, the HA K171E mutation was selected. Consistent with this observation, prior studies have demonstrated that HA K171E is selected during the passage of live-attenuated A/California/07/2009 (H1N1pdm09) vaccines in MDCK cells. Moreover, this mutation was shown to enhance viral replication in these cells (48). However, during replication in ferrets, the HA E171K mutation was selected, indicating that the mutation likely confers a selective advantage in ferrets. In contrast, while the HA 172E mutation also arose during virus rescue and passage, this mutation does not appear to confer a fitness advantage in ferrets.

In our previous work, when we assessed transmission of the 2009 pandemic H1N1 virus in chains of transmission using six trios of immunologically naïve ferrets, two mutations in HA, HA D222G and HA R223Q, were present in all six ferret trios and became enriched in five out of six trios (33). Additionally, in our previous work, we identified five other non-synonymous mutations that were present in both the RC1 and RC2 ferrets in at least one of six trios of ferrets (PB2 A84D, PB2 A184T, PB2 I562F, NP G101D, and NP D375N). In this study, we identified nine non-synonymous mutations that were associated with transmission from the Mock RC1 to RC2 ferrets (i.e., animals without immunity to influenza) in at least one chain of transmission. However, these mutations were different from those identified in our earlier studies and consisted of PB2: D253N; PA: S250A; HA: I22V, E171K, E171Q, G172E, P199Q, N211D, and E241K. Differences in mutations identified in the current study and our prior work may be attributed to differences in the viral stocks used to initiate transmission chains. In our previous study, we used a natural isolate of the A/California/07/2009 (H1N1pdm09) virus that was grown in eggs, whereas in this study, we used a recombinant strain of A/California/07/2009 (H1N1pdm09) virus. The recombinant virus was derived from reverse genetics plasmids and minimally passaged in MDCK cells. Several studies have documented differences in quasi species for viruses grown in eggs and cells (49–51).

Previous studies with the guinea pig model of influenza have evaluated the role of vaccine-induced NA immunity in preventing transmission of influenza B viruses (27, 28). In initial studies, guinea pigs were given a prime and boost vaccination with recombinant NA and adjuvant either via intranasal or intramuscular routes (27). After vaccination, animals had similar IgG antibody responses directed toward NA in the serum regardless of vaccination route. Anti-NA antibodies were detected in the nasal wash of intranasally vaccinated animals but not intramuscularly vaccinated animals, indicating a site-specific immune response was induced. Twenty-eight days post-boost vaccination, vaccinated guinea pigs were challenged with a homologous influenza B virus. These animals were then used as donors in a 1:1 donor to respiratory contact transmission study. Intramuscularly vaccinated donors transmitted the virus to 50% of their respiratory contacts, while intranasal vaccination completely blocked transmission from donors to contacts. Upon infection, all vaccinated animals shed significantly less virus than negative controls and reductions in total viral shedding (i.e., AUC) correlated with reduced transmission. When intranasally vaccinated guinea pigs were challenged with a heterologous influenza B virus and used as donors in transmission experiments, transmission was reduced to between 33-66% of contacts, but transmission was not completely blocked (27).

In follow-up studies, chains of direct contact transmission in the presence of NA immunity were evaluated in the guinea pig model. For these studies, guinea pigs were vaccinated via the intranasal route with recombinant NA combined with Poly I:C (28). Immunologically naïve donors were infected with homologous influenza B virus at doses of 10^4^ or 10^5^ plaque-forming units (PFUs), and each donor was co-housed with an NA-vaccinated guinea pig for 6 days. After 6 days, the NA-vaccinated guinea pig was then co-housed with a second NA-vaccinated animal for another 6 days. Finally, the second NA-vaccinated guinea pig was moved and co-housed with a third NA-vaccinated guinea pig, again for 6 days. As a control, chains of transmission experiments were also performed using guinea pigs vaccinated with an irrelevant NA protein. In these experiments, guinea pigs vaccinated with an irrelevant NA protein consistently transmitted the virus onward regardless of inoculation dose. Transmission was reduced between animals vaccinated with homologous NA, but not blocked completely, and was inoculation dose dependent. Specifically, chains of transmission with donor guinea pigs that were inoculated with 10^4^ plaque-forming units of influenza B virus experienced fewer transmission events overall than donors that were infected with 10^5^ PFU (28).

In our study, when intramuscular vaccination was used to induce an antibody response to NA, this immunity did not impede airborne transmission of the 2009 pandemic H1N1 virus in ferrets. However, when evaluating the effect of intramuscular vaccination against influenza B NA in guinea pigs, vaccine-induced immunity reduced but did not block airborne transmission of a homologous influenza B virus (27). It is not clear what accounts for these differences as similar vaccine approaches with recombinant NAs were used to induce antibodies against NA, and in both guinea pigs and ferrets, the 2009 pandemic H1N1 virus and influenza B viruses are readily transmitted by the airborne route (33, 52–55). In comparative studies, guinea pigs have been shown to develop a broader antibody response to influenza HA and NA compared to ferrets (56), and it is possible that this increased breadth in antibodies may be more capable of limiting viral shedding and transmission.

When comparing the results of transmission chain experiments in guinea pigs to our studies in ferrets, vaccination against influenza B NA reduced transmission in guinea pigs, while in our ferret transmission chain experiments, vaccination against NA did not disrupt transmission. This is most likely due to differences in the route of vaccination as guinea pigs were given an intranasal vaccine that induced site-specific mucosal immunity, while our intramuscular vaccine approach induced a systemic immunity response that likely did not confer mucosal immunity. In our ferret studies, we chose to use an intramuscular vaccination approach as this is the route of vaccination used for licensed inactivated influenza vaccines, and there are currently no licensed intranasal adjuvants for humans. However, in future studies, it will be valuable to assess the impact of intranasal vaccination against influenza A NA on sequential airborne transmission in ferrets.

Another limitation of our study is that only immunity to N1 NA was evaluated in the context of infection with the 2009 pandemic H1N1 virus. Future work is warranted to evaluate if N2 immunity would impair the transmission of a human H3N2 influenza virus. In our prior sequential transmission studies, the A/Texas/50/2012 (H3N2) virus replicated to lower titers in ferrets than the 2009 pandemic H1N1 virus. Thus, vaccine-induced N2 immunity may result in sufficient reductions in viral shedding to reduce transmission in ferrets (33). Furthermore, it will be important to assess the role of shorter exposure times for both the DI and RC1, as well as RC1 and RC2 ferrets, and the role of vaccine-induced immunity to other antigens, such as HA, or prior infection on sequential airborne transmission.

Collectively, we demonstrate that ferrets vaccinated against NA were susceptible to infection by respiratory contact with donor ferrets infected with the 2009 pandemic H1N1 virus. Vaccine-induced NA immunity was able to reduce viral replication in the nose and accelerate viral clearance when NA-vaccinated ferrets became infected. Despite these reductions, vaccine-induced NA immunity was not sufficient to prevent onward airborne transmission to secondary respiratory contacts. These studies show that NA immunity is not sufficient to prevent infection by the 2009 pandemic H1N1 virus during continuous respiratory contact exposure, and NA immunity was not sufficient to disrupt chains of airborne transmission. While no effects were observed on transmission, these studies emphasize the ability of immunity toward NA to limit viral replication, and further development of NA as a vaccine antigen is warranted (16).

## MATERIALS AND METHODS

### Cells

MDCK cells, London line, were obtained through the International Reagent Resource, Influenza Division, World Health Organization (WHO) Collaborating Center for Surveillance, Epidemiology and Control of Influenza, Centers of Disease Control and Prevention (CDC), Atlanta, GA, USA. Cells were maintained in a humidified incubator at 37°C with 5% CO_2_ in Dulbecco’s Modified Eagle Medium (Cytvia) with fetal bovine serum (10%), HEPES (25 mM), L-glutamine (2 mM, Corning), and 1% antibiotic-antimycotic (Gibco).

### Viruses and viral titration methods

Recombinant A/California/07/2009 (H1N1pdm09) virus was generated using bi-directional reverse genetics plasmids. The virus was passaged twice in MDCK cells to generate a stock, and the virus stock was titrated on 24 well plates to determine the tissue culture infectious dose 50% (TCID50) as previously described (57). Nasal wash samples were titrated on MDCK cells in 96-well plates to determine the TCID50/mL (57). The virus and nasal wash samples were stored at −80°C until use.

### Vaccination to induce NA antibodies

Twenty-three-week-old male and female ferrets (Triple F Farms, Sayre, PA) were screened by hemagglutinin inhibition assay and confirmed serologically negative for currently circulating influenza A viruses. Equal numbers of male and female ferrets were divided into two groups and received a mock vaccination or vaccination with NA protein. Ferrets that received NA protein were vaccinated intramuscularly (i.m.) with 50 µg of baculovirus-expressed A/California/04/2009 N1 NA recombinant protein (Sino Biological Cat No.11058-V08B) generated with vasodilator-stimulated phosphoprotein tetramerization domain at the N-terminus. The recombinant NA protein (specific activity >8,000 pmoles/min/μg using 2'-(4-Methylumbelliferyl)-α-D-N-acetylneuraminic acid substrate and >90% purity by SDS-PAGE) was mixed 1:1 with oil-in-water adjuvant, Sigma Adjuvant System (Sigma Aldrich). Mock-vaccinated animals were vaccinated with adjuvant mixed with PBS. Ferrets received a primary vaccination and two boost vaccinations 28 days apart. Each vaccine had a total volume of 0.5 mL, and 0.250 mL was administered in each hind limb. Blood collection was performed every 14 days, and serum was isolated and stored at −20°C. At 90 days post-primary vaccination (28 days after the second boost/third vaccination), the vaccinated ferrets served as respiratory contacts in airborne transmission experiments.

### Transmission chain experiments

Transmission chain experiments were performed as previously described with modifications to use vaccinated ferrets as RC1 animals (33). Groups (*n* = 6/group) of 23-week-old age and sex-matched animals were used as DI and RC2 animals. Groups of six DI animals were anesthetized with a mixture of ketamine (20 mg/kg), xylazine (2 mg/kg), and atropine (0.05 mg/kg), and intranasally inoculated with 1 mL of 10^6^ TCID50/mL of recombinant A/California/07/2009 (H1N1 pdm09) virus. DI ferrets were paired with mock (*n* = 6) or NA vaccinated (*n* = 6) RC1 animals 24 hours post-infection in transmission cages. Nasal wash samples were collected from DI animals every other day for 9 days by instilling 1 mL of PBS into the animal’s nasal passage and inducing sneezing by gently stimulating the nose of the animals. The expelled liquid was collected onto a petri dish. The petri dish was rinsed with 1 mL of PBS and aliquoted and stored at −80°C. In the RC1 animals, nasal wash samples were collected daily to detect viral RNA in the nasal wash. RNA was extracted from 0.140 mL of nasal wash using the QIAmp Viral RNA mini kit (Qiagen). Levels of RNA were quantified using the Superscript III Platinum One-Step qRT-PCR system (Invitrogen) and influenza A primers and probe (Integrated DNA Technologies) according to the CDC protocol published by the WHO (58). A Ct value of less than 35 was considered positive. qRT-PCR was performed on a Roche Light Cycler 480 II. Once an RC1 animal was determined to be infected, it was transferred to a new transmission cage where it served as the donor animal to a naïve RC2. Animals were nasal washed every other day for 14 days in the second round of transmission. Twenty-one days post-infection of the DI ferret, animals were deeply sedated, and a terminal blood collection was performed via cardiac puncture. Animals were then euthanized via overdose with sodium pentobarbital. All animal studies were conducted in accordance with the Penn State University Institutional Animal Care and Use Committee Protocol# 2018000250.

### NA antibody ELISA

ELISA was performed to quantify NA antibodies in ferret serum. Ninety-six well plates (Nunc) were coated with 100 ng/well of recombinant N1 (A/California/07/2009) NA protein (Sino Biological) in sodium bicarbonate buffer and incubated overnight at 4°C. The next day, plates were blocked with PBST (0.05% Tween) with goat serum (3%) and skim milk powder (5%) for 2 hours at room temperature. Plates were washed with PBST and then incubated with serial fourfold dilutions of heat-inactivated serum (starting at 1:40) in PBST with 1% skim milk powder for 2 hours at room temperature. Plates were washed and incubated with horseradish peroxidase (HRP)-conjugated anti-ferret goat IgG antibody at a 1:1,000 (Alpha Diagnostic) dilution for 2 hours at room temperature. *o*-Phenylenediamine dihydrochloride (OPD) substrate solution (Sigma) was added to all wells after 6× PBST washes and incubated for 10 minutes in the dark. The reaction was stopped by adding 3M HCl, and optimal density was measured at 490 nm on a SpectraMax iD3 plate reader (Molecular Devices). An absorbance value greater than three times the SD of the mean day 0 value was considered positive.

### Enzyme-linked lectin assay

Enzyme-linked lectin assays were performed as previously described (59). Ninety-six-well plates (Nunc) were coated with 25 µg/µL fetuin in PBS and stored at 4°C overnight. In a separate dilution plate, heat-inactivated ferret serum (starting dilution of 1:160) was serially diluted and mixed with an inactivated recombinant H6N1 virus containing the HA from A/turkey/Massachusetts/3740/1965 (H6N2), NA from A/California/07/2009 (H1N1pdm09), and the remaining six genes from the A/Puerto Rico/8/1934 (H1N1) virus diluted (1:5000) in PBS with calcium and magnesium, bovine serum albumin (BSA, 1.0%), and Tween-20 (0.05%). Fetuin-coated plates were washed with PBST (0.05% tween), and the serum-virus mixture was transferred from the dilution plate to the fetuin-coated plates. Plates were incubated overnight at 37°C. The following day, plates were washed with PBST and incubated with biotinylated lectin from *Arachis hypogaea* (PNA-Bio, Sigma) diluted 1:500 in PBS with calcium and magnesium and BSA (1.0%) for 2 hours at room temperature in the dark. After washing, streptavidin-HRP (1:500, Millipore) in PBS with calcium and magnesium and BSA (1.0%) was added and incubated for 1 hour at room temperature in the dark. OPD substrate solution (Sigma) was added to all wells, and 1 N sulfuric acid was added to stop the reaction after 10 minutes. Optical density was measured at 490 nm on a SpectraMax iD3 plate reader (Molecular Devices), and end point titers were calculated by determining the serum dilution that resulted in at least 50% inhibition of the maximum signal.

### Hemagglutination inhibition assays

Serum isolated from ferrets in transmission chain experiments was treated with receptor-destroying enzyme and heat-inactivated. Hemagglutination inhibition titers were then determined using turkey red blood and recombinant A/California/07/2009 (H1N1pdm09) virus as previously described (60).

### Whole-genome sequencing

Nasal wash samples with viral titers over 10^2^ TCID_50_/mL were subjected to whole-genome sequencing. Viral RNA was extracted from 140 µL of nasal wash samples collected from donor and recipient ferrets using the QIAmp Viral RNA Mini Kit (QIAGEN) according to the manufacturer’s instructions. RNA underwent RT-PCR using SuperScript III One-Step RT-PCR System with Platinum Taq High Fidelity (Invitrogen) and influenza universal primers. PCR clean-up was performed using AMPure XP beads (Beckman), and samples were normalized and libraries prepared using the Nextera XT DNA Library Preparation Kit (Illumina) and IDT for Illumina Nextera DNA Unique Dual Index Set. Again, samples underwent PCR clean-up using AMPure XP beads, and libraries were pooled at equal molarities after concentration was determined using Qubit dsDNA HS Assay Kit (Invitrogen). Fragment size was then determined using TapeStation High Sensitivity, and size selection was performed on the pooled library using SPRISelect Beads (Beckman). The library was normalized and diluted to a final concentration of 14 pM. Sequencing was performed on an Illumina MiSeq using the MiSeq Reagent Kit v3 (600 cycles). All samples were processed in duplicate. Raw sequencing data from the stock inoculum samples were first trimmed (Trimmomatic v0.39) and aligned (BWA v0.7.17) to the full-length A/California/07/2009 reference sequence (CY121680, CY121681, CY121682, CY121683, CY121684, CY121685, CY121686, and CY121687). Using timo (v1) and a read depth requirement of 5×, stock consensus sequences were generated for each gene segment by pulling either the full coding sequence (segments 1–6) or the sequence beginning at the first start codon and ending at the last stop codon (segments 7 and 8). Replicate stock consensus sequences were confirmed to be identical before trimming (Trimmomatic v0.39) and aligning (BWA v0.7.17) the raw sequencing data for each ferret sample to its respective stock inoculum consensus. Duplicate reads were marked and removed (PicardTools v.2.17.11) before calling major and minor variants using timo (v1, https://github.com/GhedinLab/timo) and iVar (v.1.3.1) (61). Major variants were called at a relative frequency of 50% or greater. Minor variants were called at a frequency of 1%–49.9%. Variants used for analyses had to be in both sequencing replicates at positions with ≥10× read depth and relative frequencies ≥1%. All variant figures show the average relative frequency of the variant in both sequencing replicates. Data visualization was carried out in R (v.4.2.3). Raw sequencing data are available under Bioproject PRJNA1063699. Variant tables and code used for data visualization are available on GitHub at https://github.com/GhedinSGS/NA_Vax_Ferret_Transmission.

### Statistical analyses

Area under the curve (Fig. 4B) and average viral titers over time for Mock RC1 and NA RC1 animals (Fig. 4C) were tested for normality using a Kolmogorov-Smirnov test. AUC values were compared using a one-way ANOVA with post-hoc Tukey’s test, and average viral titers over time were compared by two-way ANOVA with repeated measures. *P* < 0.05 was considered significant. Peak viral titers and duration post-contact at which ferrets began shedding virus were compared by Kruskal-Wallis test and Mann-Whitney U tests, respectively. Prism GraphPad v10 was used for all statistical analyses and to calculate area under the curve.

## SUPPLEMENTAL MATERIAL

Supplemental Tables and Figures mbio.02161-24-s0001.pdf Table S1 and Figures S1-S5.

ASM does not own the copyrights to Supplemental Material that may be linked to, or accessed through, an article. The authors have granted ASM a non-exclusive, world-wide license to publish the Supplemental Material files. Please contact the corresponding author directly for reuse.
