# Supplementary material for: Vaccine-induced NA immunity decreases viral shedding, but does not disrupt chains of airborne transmission for the 2009 pandemic H1N1 virus in ferrets
Source: mBio. 2024 Sep 9;15(10):e02161-24. doi: 10.1128/mbio.02161-24 (PMC11481891; doi:10.1128/mbio.02161-24)
Supplement: Supplemental Tables and Figures — Table S1 and Figures S1-S5. [file mbio.02161-24-s0001.pdf]

SUPLEMENTAL MATERIALS

Supplemental Tables:

Table S1: Changes in body temperature during ferret transmission chain experiments using Mock and NA vaccinated RC1 ferrets.

Supplemental Figures:

Figure S1: Percent weight loss in ferret transmission chain experiments using Mock and NA vaccinated RC1 ferrets. Panels (A) and (B) display percent of original body weight (y-axis) for chains of transmission with Mock RC1 and NA RC1 animals across days post-infection of the DI (x-axis). Magenta, green, and blue lines represent the percent weight loss for the DI, Mock RC1, and RC2 ferrets, respectively. Panel (C) shows the peak percent weight loss (y-axis) during the transmission study in each individual animal, separated by group (x-axis). Black bar denotes the mean and error bars denote standard error of the mean.

Figure S2: Non-synonymous mutations in the PB2 protein identified during sequential transmission. The relative frequency (y-axis) of PB2 SNVs across the days of the experiment (x-axis). The displayed mutations were selected based on the criteria of being non-synonymous and occurring at frequencies of 1% (0.01) or higher in at least two samples, one of which had to be an RC1 sample collection. Data are grouped by the six mock vaccinated and six NA vaccinated transmission chains (t1-t6, across) and

variants (down). The S column heading denotes the frequency of a variant in the viral stock used for infection. The dashed horizontal line represents 1% (0.01) across all plots. The point shape depicts if the variant is ≥1% (0.01, circle) or <1% (0.01, "X") in the sample. The color of each point and line indicates the ferret in each transmission pair. DI = directly infected, RC1 = Respiratory Contact 1, RC2 = Respiratory Contact 2. Empty plots indicate the mutation was not found in any of the 6 transmission chains for the given vaccination group.

Figure S3: Non-synonymous mutations in the PB1 protein identified during sequential transmission. The relative frequency (y-axis) of PB1 SNVs across the days of the experiment (x-axis). The displayed mutations were selected based on the criteria of being non-synonymous and occurring at frequencies of 1% (0.01) or higher in at least two samples, one of which had to be an RC1 sample collection. Data are grouped by the six mock vaccinated and six NA vaccinated transmission chains (t1-t6, across) and variants (down). The S column heading denotes the frequency of a variant in the viral stock used for infection. The dashed horizontal line represents 1% (0.01) across all plots. The point shape depicts if the variant is ≥1% (0.01, circle) or <1% (0.01, "X") in the sample. The color of each point and line indicates the ferret in each transmission pair. DI = directly infected, RC1 = Respiratory Contact 1, RC2 = Respiratory Contact 2. Empty plots indicate the mutation was not found in any of the 6 transmission chains for the given vaccination group.

Figure S4: Non-synonymous mutations in the PA protein identified during sequential transmission. The relative frequency (y-axis) of PA SNVs across the days of the experiment (x-axis). The displayed mutations were selected based on the criteria of being non-synonymous and occurring at frequencies of 1% (0.01) or higher in at least two samples, one of which had to be an RC1 sample collection. Data are grouped by the six mock vaccinated and six NA vaccinated transmission chains (t1-t6, across) and variants (down). The S column heading denotes the frequency of a variant in the viral stock used for infection. The dashed horizontal line represents 1% (0.01) across all plots. The point shape depicts if the variant is ≥1% (0.01, circle) or <1% (0.01, "X") in the sample. The color of each point and line indicates the ferret in each transmission pair. DI = directly infected, RC1 = Respiratory Contact 1, RC2 = Respiratory Contact 2. Empty plots indicate the mutation was not found in any of the 6 transmission chains for the given vaccination group.

Figure S5: Non-synonymous mutations in the NP, M1, M2, NS1, NS2 proteins identified during sequential transmission. The relative frequency (y-axis) of (A) NP, (B) M1, (C) M2, (D) NS1, and (E) NS2 SNVs across the days of the experiment (x-axis). The displayed mutations were selected based on the criteria of being non-synonymous and occurring at frequencies of 1% (0.01) or higher in at least two samples, one of which had to be an RC1 sample collection. Data are grouped by the six mock vaccinated and six NA vaccinated transmission chains (t1-t6, across) and variants (down). The S column heading denotes the frequency of a variant in the viral stock used for infection. The dashed horizontal line represents 1% (0.01) across all plots. The point

shape depicts if the variant is ≥1% (0.01, circle) or <1% (0.01, "X") in the sample. The color of each point and line indicates the ferret in each transmission pair. DI = directly infected, RC1 = Respiratory Contact 1, RC2 = Respiratory Contact 2. Empty plots indicate the mutation was not found in any of the 6 transmission chains for the given vaccination group.

Table S1. Changes in body temperature during ferret transmission chain experiments using Mock and NA vaccinated RC1 ferrets

Immune Status of RC1	Ferret Trio	Group	Ferret Number	Baseline	Maximum	Change from Baseline	Day p.i. of DI or Alt day
Mock	1	**DI**	2457	38.3	39.1	0.8	7 p.i.
		**RC1**	1128	38.9	39	0.1	6 p.i./3 Alt
		**RC2**	2461	38.6	39.2	0.6	10 p.i./7 Alt
	2	**DI**	2458	39.5	39.5	0	0 p.i.
		**RC1**	1132	38.9	39.7	0.8	8 p.i./5 Alt
		**RC2**	2462	38.9	39.5	0.6	10 p.i./7 Alt
	3	**DI**	2459	38.5	38.5	0	0 p.i.
		**RC1**	1133	39	39	0	1 p.i./0 p.c.
		**RC2**	2464	38.9	38.9	0	4 p.i./1 Alt
	4	**DI**	2465	39.1	39.1	0	0 p.i.
		**RC1**	1117	38.2	38.5	0.3	5 p.i./1 Alt
		**RC2**	2468	38.5	39.5	1	11 p.i./7 Alt
	5	**DI**	2466	38.5	39.4	0.9	5 p.i.
		**RC1**	1118	38.4	39.7	1.3	4 p.i./1 Alt
		**RC2**	2470	38.3	39	0.7	8 p.i./5 Alt
	6	**DI**	2467	38.2	38.4	0.2	9 p.i.
		**RC1**	5813	38.9	40	1.1	5 p.i./1 Alt
		**RC2**	2472	37.8	40	2.2	9 p.i./5 Alt
NA	1	**DI**	2901	39.1	40.1	1	7 p.i.
		**RC1**	5814	39	39	0	0 p.i.
		**RC2**	2904	37.1	39.2	2.1	12 p.i./9 Alt
	2	**DI**	2902	38.6	38.8	0.2	9 p.i.
		**RC1**	1115	38.8	39.5	0.7	4 p.i./1 Alt
		**RC2**	2905	37.9	39.1	1.2	10 p.i./7 Alt
	3	**DI**	2903	39.1	39.3	0.2	1 p.i.
		**RC1**	1116	39.6	39.6	0	0 p.i.
		**RC2**	2906	38.6	38.6	0	9 p.i./5 Alt
	4	**DI**	3109	38.6	39.3	0.7	5 p.i./2 Alt
		**RC1**	2812	39.8	39.8	0	0 p.i.
		**RC2**	3120	38.7	38.9	0.2	11 p.i./8 Alt
	5	**DI**	3110	38.3	39.3	1	3 p.i.
		**RC1**	1122	38.9	39.6	0.7	4 p.i./1 Alt
		**RC2**	3122	38.1	38.8	0.7	10 p.i./7 Alt
	6	**DI**	3111	39.3	39.3	0	0 p.i.
		**RC1**	1129	39.2	39.3	0.1	12 p.i./9 Alt
		**RC2**	3123	38.2	39.2	1	8 p.i./5 Alt

Notes:
p.i. denotes day post-infection of donor (DI) ferret
p.c. dnotes days post-contact for RC1 with DI
Alt denotes Alternate day which is the day after ferret RC1 was paired with RC2

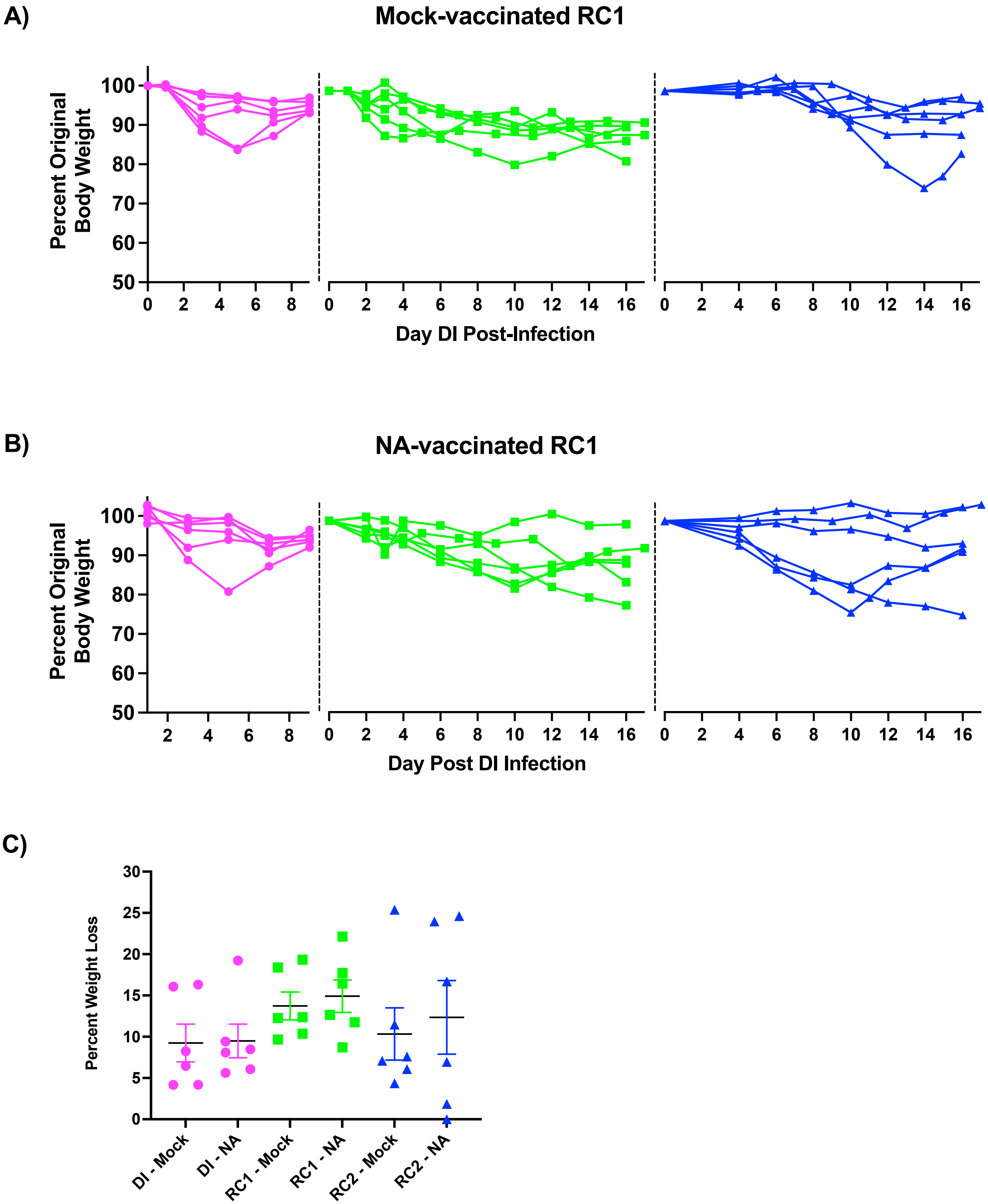
A)
Mock-vaccinated RC1
Percent Original Body Weight
Day DI Post-Infection
B)
NA-vaccinated RC1
Percent Original Body Weight
Day Post DI Infection
C)
Percent Weight Loss
DI - Mock
DI - NA
RC1 - Mock
RC1 - NA
RC2 - Mock
RC2 - NA

Figure S1

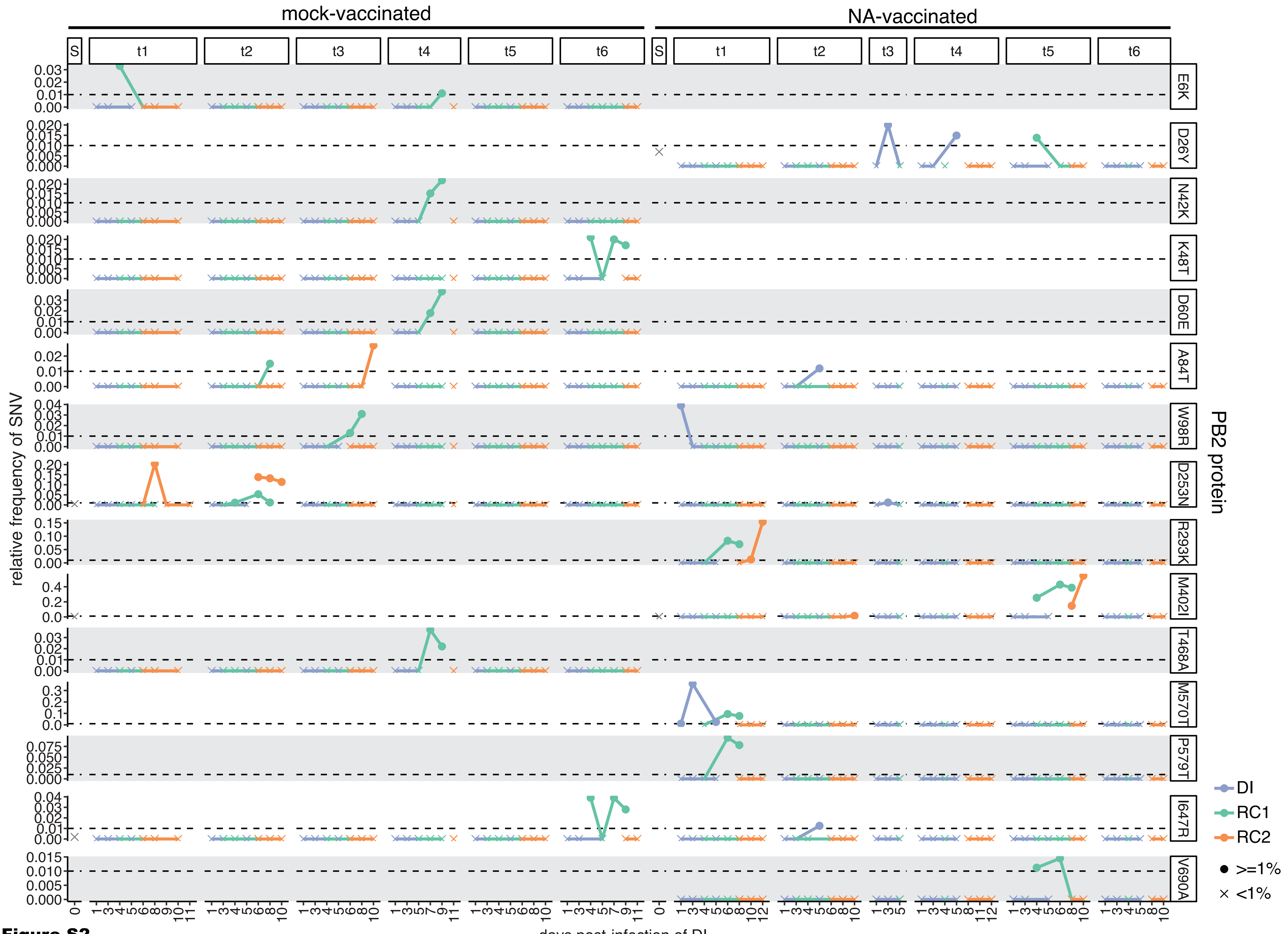

mock-vaccinated
NA-vaccinated
S
t1
t2
t3
t4
t5
t6
E6K
D26Y
N42K
K48T
D60E
A84T
W98R
D253N
R293K
M402I
T468A
M570T
P579T
I647R
V690A
PB2 protein
relative frequency of SNV
days post-infection of DI
DI
RC1
RC2
>=1%
<1%

Figure S2

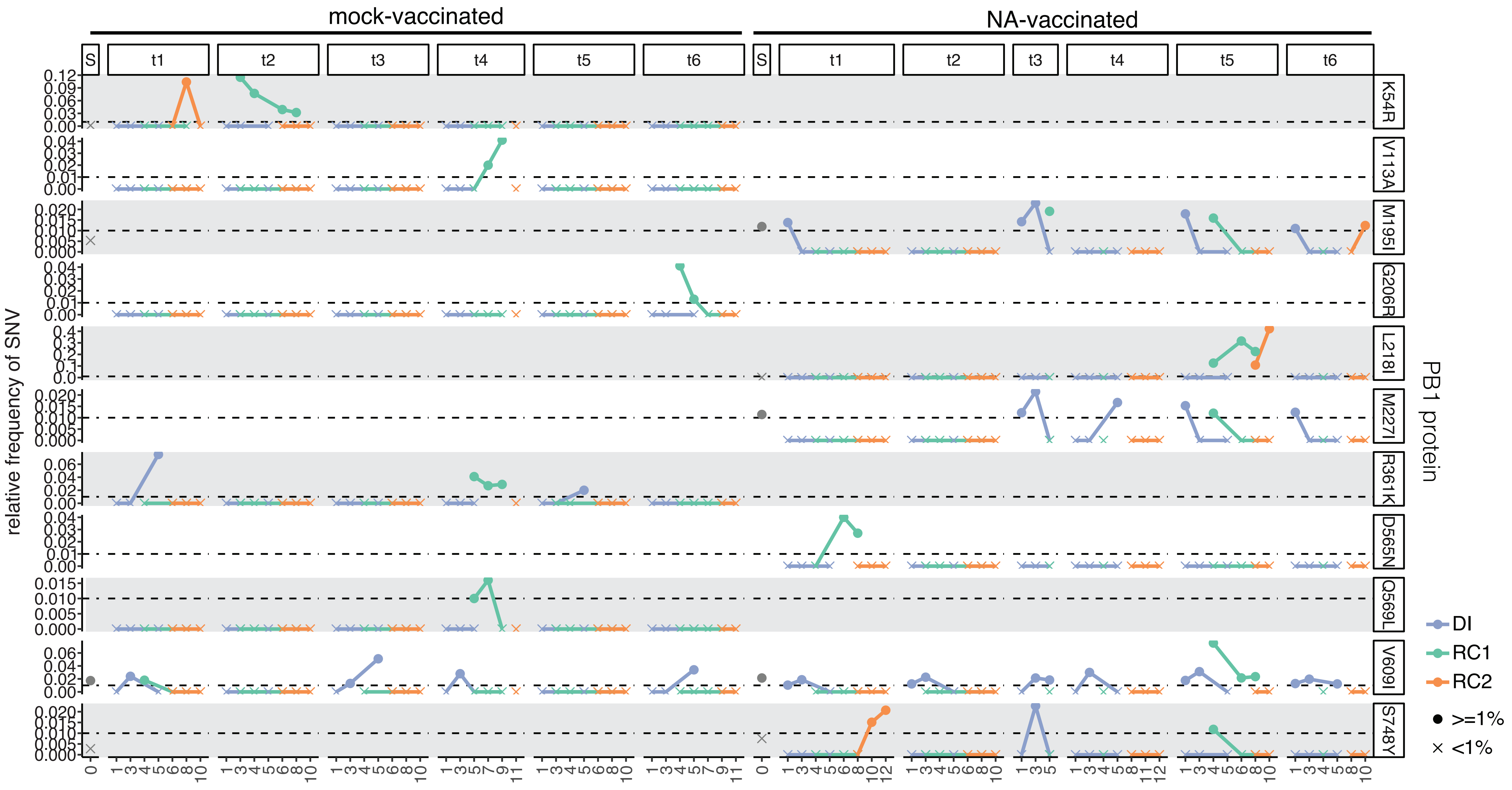

mock-vaccinated
NA-vaccinated
S
t1
t2
t3
t4
t5
t6
K54R
V113A
M195I
G206R
L218I
M227I
R361K
D565N
Q569L
V609I
S748Y
PB1 protein
relative frequency of SNV
days post-infection of DI
DI
RC1
RC2
>=1%
<1%

Figure S3

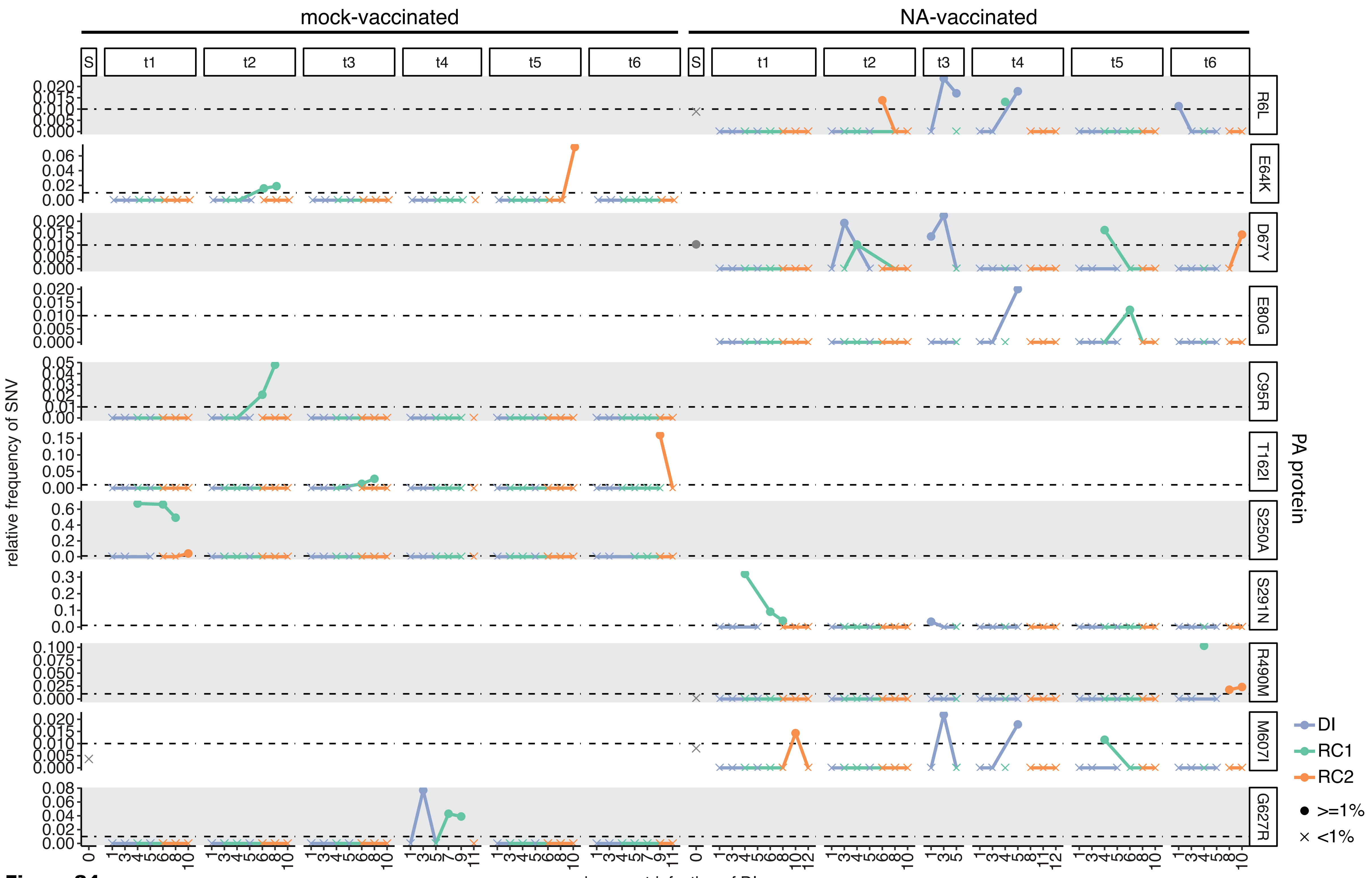
mock-vaccinated
NA-vaccinated
S
t1
t2
t3
t4
t5
t6
R6L
E64K
D67Y
E80G
C95R
T162I
S250A
S291N
R490M
M607I
G627R
PA protein
relative frequency of SNV
days post-infection of DI
DI
RC1
RC2
>=1%
<1%

Figure S4

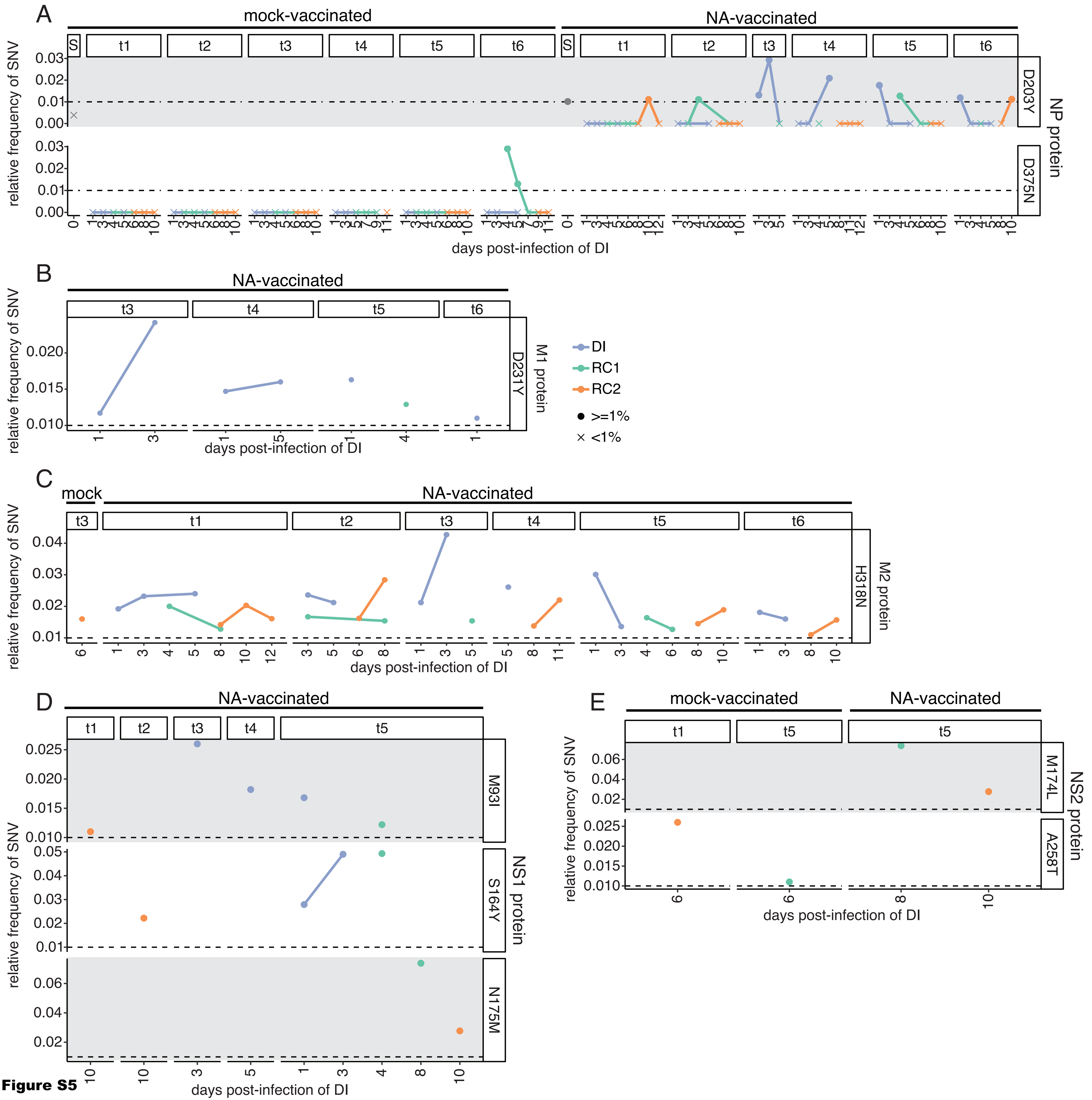

A
mock-vaccinated
NA-vaccinated
S
t1
t2
t3
t4
t5
t6
relative frequency of SNV
D203Y
D375N
NP protein
days post-infection of DI
B
NA-vaccinated
M1 protein
D231Y
DI
RC1
RC2
>=1%
<1%
C
mock
M2 protein
H318N
D
NS1 protein
M93I
S164Y
N175M
E
NS2 protein
M174L
A258T

Figure S5
